# The impact of neighborhood mental health on the mental health of older adults

**DOI:** 10.1186/s12889-023-16263-w

**Published:** 2023-07-14

**Authors:** Rengui Gong, Dongping Xia, Zan Hu, Yangming Hu

**Affiliations:** 1 School of Public Management and Law, Anhui University of Technology, Ma’anshan, China; 2 College of Public Administration and law, Hunan Agricultural University, Changsha, China; 3 The Affiliated Changsha Central Hospital, Hengyang Medical School, University of South China, Changsha, China

**Keywords:** Mental health, Neighborhood mental health, Older adults, China community

## Abstract

**Background:**

In this article, we use cross-sectional data obtained from the 2018 China Health and Aging Tracking Survey (CHARLS) to examine the impact of neighborhood mental health at the community level on the mental health of older adults aged 60 years and older.

**Methods:**

NMH is the average mental health of older adults in the same community, excluding the older adults themselves. The explained variable mental health in this paper was measured using the simple CES-D depression scale. The mediating variables were social connectedness, social participation and social inclusion, and the instrumental variables were physical exercise and amusement. regression analysis was conducted using OLS regression models, two-stage least squares (IV-2SLS) instrumental variables to address the two-way causality of NMH and MH, and KHB decomposition was used to investigate the effect mechanism.

**Results:**

Baseline regressions showed that the neighborhood mental health effect positively influenced the mental health of older adults (Coef. = 0.356, 95% CI 0.315,0.397). The neighborhood mental health effect estimated by IV-2SLS (Coef. = 0.251, 95% CI 0.096,0.405) was higher than the OLS regression, indicating endogeneity. The mediated effects of KHB showed total (Coef. = 0.356, 95% CI 0.314,0.398), direct (Coef. = 0.281, 95% CI 0.232,0.330), and indirect effects (Coef. = 0.075, 95% CI 0.049,0.101). While the total effect was 1.266 times higher than the direct effect, 21.03% of the total effect came from mediating variables.

**Conclusions:**

First, the neighborhood mental health effect has a positive impact on the mental health of older adults, but there are heterogeneous differences based on gender, age, and place of residence. Second, the results of the IV-2SLS estimation showed that the effect of the neighborhood mental health effect was underestimated if endogenous problems were not controlled for. Third, the effect of neighborhood mental health on older adults' mental health was tested to be stable. Moreover, social connectedness, social participation, and social interaction are important mediating mechanisms for the effect of neighborhood mental health on older adults' mental health. This study provides new perspectives and ideas for an in-depth understanding of the mental health of older adults in the context of social transformation in China.

## Introduction

The older population, which was aged 60 or above in the Chinese Mainland, reached 267 million, accounting for 18.9% of the total population by December 2021, and the situation of population aging is serious [1]. Moreover, the average life expectancy in China is 77 years, but the healthy life expectancy is only 68.7 years, implying that more than 180 million older adults will have to live with chronic diseases for more than 8 years [2]. The huge base of the older population and limited medical and health facilities have a serious mismatch between supply and demand. To alleviate health problems caused by an aging population, the Chinese government issued the 14th Five-Year Plan for Healthy Aging [3]. This plan is actively exploring ways to alleviate the shortage of resources for seniors and promote healthy living for seniors. According to the World Health Organization (WHO), health is affected by four main factors: genetic endowment (15%), environment (17%), medical and sanitary conditions (8%), and personal living habits and living behaviors (60%). Among them, the environment, medical and sanitary conditions, personal living habits, and behaviors all have a certain relationship with the neighborhood environment [4]. Especially in China's "acquaintance society" which emphasizes "differential pattern," the neighborhood effect has a more intimate impact on people's health. WHO (2002) Active aging: A policy framework expanded the perspective of attention to the psychological level of older adults. Nowadays, Chinese people pay more attention to both mental and physical health. Friendly neighborhood relations are once again recognized and pursued. There have been relevant studies on the influence of neighborhood effects on the physical health of the older adults, but only a few studies exist on the impact of neighborhood effects on the mental health of the older adults, particularly significant changes of the neighborhood relationship in China. Therefore, based on the background of China's "acquaintance society", this paper presents the influence of neighborhood effect on the mental health of the older adults in China, which is of great practical significance to the construction of harmonious community, and also provides Chinese cases for the study of neighborhood effect theory.

## Literature review

Wilson first proposed a neighborhood effect on the behavior of the American lower class [5]. He argues that mainstream society excludes America's ghettos, and that the degree of neighborhood segregation is linked to unemployment rates in the lower class. Neighborhood effects are the effects of many people with certain characteristics living together on residents' employment opportunities, health status, life attitudes, behaviors, and education levels of the next generation [6]. The social effects of this gathering of specific groups have also been widely studied, with particular attention paid to the effects of neighborhoods on the health status of residents. Meanwhile, scholars have achieved more results on the health effects of neighborhood effects [7–12]. In the study of neighborhood effects, the neighborhood environment is usually divided into the neighborhood-built environment and the neighborhood social environment [13]. The built environment mainly refers to the man-made physical environment [14]. Research shows that area disadvantage has a psychological impact on people [15]. Community green space, walkability index and increased street connectivity all correlate with health [16–18]. And the community-built environment has been shown to be more associated with overall cognition, memory and dementia in older adults [19]. Not only that, but the availability of recreational facilities affects physical activity, obesity and diabetes prevalence [20]. Neighborhood social environment mainly refers to socio-cultural features [13]. Studies have shown that poverty, low income, unemployment, and reduced access to public health insurance not only significantly increase mental health disorders [21, 22]. And suffering neighborhood deprivation in the course of life has an impact on one's health and well-being [23]. In addition, scholars have found that living in a positive neighborhood social environment promotes mental health. Residents living in neighborhoods with higher social cohesion, neighborhood attachment, and social contact have a higher sense of belonging, less loneliness, and a lower prevalence of depression, which is beneficial to people's mental health [24].

Scholars have conducted more comprehensive and in-depth studies on the mental health of older adults, such as the impact of personal circumstances (gender, age, marriage, exercise, ADL or IADL) on mental health. Older adults without a partner (single, unmarried, divorced or widowed) have been shown to be more likely to suffer from depression than those with a partner because they feel more secure with their partner, which improves their health [7, 25], or any ADL or IADL limitations were significantly associated with depression and decreased life satisfaction [26]. In addition to personal characteristics, more research results have been obtained on the influence of socioeconomic factors (e.g., health care, lifestyle, income level, education level, and living environment) on mental health. For example, higher socioeconomic status has a significant effect on the health of older adults [27]. Conversely, lower socioeconomic status can have a negative impact on health as it leads to more negative emotions or potential stress in life [28].

This study explores the effect of neighborhood mental health on the mental health of the older adults in the context of China's traditional acquaintance society, which is gradually transforming into a stranger society. It analyzes the internal mechanism of the effect of neighborhood mental health, which provides an important theoretical reference for addressing the mental health problems of the older adults. This study provides new perspectives and ideas for a deeper understanding of the mental health problems of the older adults in the context of China's social transformation.

## Materials and methods

### Study sample

The data studied in this paper is based on a long-term follow-up survey project organized by Peking University: China Health and Retirement Longitudinal Study. This project is a multidisciplinary and interdisciplinary comprehensive survey, which started in 2011 and has been carried out for four periods. CHARLS 2018 is the latest data available. The project involved 11,797 households and 20,284 residents in 150 counties in 28 provinces across China. The survey content covers the basic demographic characteristics, health conditions and functions, medical insurance, work and retirement, family situation, and other information, including rich information about individual and community characteristics. The relationship between the neighborhood effect and the health of the older adults can be explored by analyzing the data of individuals and the community. In terms of data processing, first, delete the samples under 60 years old in the CHARLS 2018 survey to ensure that the research objects are elderly individuals aged 60 years and above. Second, eliminate the samples of missing key information and abnormal values, and finally, retain 7713 mixed cross-section samples of elderly individuals in 126 prefecture-level cities for empirical analysis.

### Variables

#### Explained variable

Mental health. The explanatory variable in this paper is the mental health of the older adults, and the CHARLS 2018 Family Questionnaire is a simple CES-D depression scale used to measure the mental health of the older adults. The 10 questions on feelings and behaviors are: whether they are bothered by small things, whether they can concentrate, whether they are depressed, whether they find it hard to do anything, whether they are hopeful about the future, whether they are afraid, whether they sleep well, whether they are happy, whether they are lonely, and whether they feel they can continue to live. Respondents could choose to answer these 10 questions rarely or none of the time, some, occasionally, and most or all of the time. In the processing of the data, respondents' answers are assigned values from 4 to 1, corresponding to a scale from positive to negative. The scores from the 10 responses are then aggregated and divided by 10. The resulting score reflects the mental health of older adults and falls within the range [1, 4], where 1 indicates the worst mental health and 4 indicates the best mental health.

#### Main explanatory variable

Neighborhood mental health. The neighborhood scope studied in this report is mainly at the community level because the community is the closest activity place for older adults in their daily lives. If the neighborhood scope is too expanded, the neighborhood effect is weakened, making it difficult to observe the effects on the mental health of the neighborhood. We defined NMH as the average level of mental health of older adults in the community *I*, excluding older adults individuals *i*, after considering Mansky's social interaction effect model and the practice of Ling et al. [29, 30]. The specific calculation of NMH is shown in Eq. 1:

1 $${NMH}_{i}=\left({MH}_{I}-{MH}_{i}\right)/\left(n-1\right)$$

Within the explained variable section, the mental health of older adult *i* (${MH}_{i}$) was measured using the simple CES-D depression scale. Meanwhile, using the community coding information provided by CHARLS 2018, we can calculate the total number of older adults (*n*) living in the same community, as well as the cumulative sum of their mental health (${MH}_{I}$). Then, by applying Eq. 1, the neighborhood health effect of older adult *i* (${NMH}_{i}$) can be calculated.

#### Instrumental variables

Physical exercise and amusement of other older adults. In order to exclude the existence of a causal relationship between the mental health of older adults and the NMH, the instrumental variable estimation method (IV) was introduced. Many scholars, such as Manski [31], Card & Krueger [32], Moffitt & Comments [33], Paul Schultz [34], Bobonis & Finan [35], often use higher-level aggregation data as instrumental variables to address the endogeneity problem in peer effect studies. This study is also constructed in this way, using neighborhood physical exercise and neighborhood leisure activities as instrumental variables at the community level, a higher level than the individual elderly. Previous studies have shown that physical exercise has a significant impact on mental health [20, 36, 37], but it only affects the mental health of certain older adults who participate in physical exercise, while the impact on the mental health of other older adults is very small. Neighborhood physical exercise in this study refers to the average physical exercise of other older adults in the community, except for specific older adults, and only affects NMH, not the specific older adults' MH directly. In other words, neighborhood physical exercise affects MH only through NMH, which satisfies the two core conditions of instrumental variables. There are also many studies on the effects of recreational activities on mental health [38–40], and the related discussions are similar to those mentioned above. From a theoretical perspective, we can confidently conclude that the instrumental variables used in this article are appropriate.

#### Mediating variables

Based on social interaction theory, we constructed three mediating variables, namely social contact, social participation, and social interaction, from three dimensions of social interaction: breadth, frequency, and depth, respectively. Surfing the Internet is a proxy variable for social contact and can reflect the size of the social interaction field. Volunteer activities is a proxy for social participation and may reflect the frequency of social interaction. Gift money is a proxy for social interaction and may reflect the depth of social interaction.

#### Control variables

The mental health of older adults is affected by many factors. The following variables are selected as control variables based on the relevant existing research [41–43]. First, some personal factors, including gender, age, marital status, educational attainment, and habitation [42]. Second, there are functional limitations, including economic status, ADL, and IADL. For example, the economic status of older adults significantly affects their quality of life and mental state [44]. Therefore, the present research uses the total income of older adults (including subsidies and children's economic support) as the proxy variable of economic factors. Third, family care is essential, and family support is the primary factor in providing for older adults [45]. Thus, three variables are included in the model: the number of children, children's economic support, and children's accompanying time. Finally, we believe that public policy must also be considered. The diseases caused by human aging significantly impact the mental health of older adults as they age. Good public medical facilities can effectively alleviate pain caused by physical functions [46]; thus, we chose two indicators to assess public medical services: medical service satisfaction and paid family doctor service.

Table 1 Depicts the meaning and descriptive statistics of the main variables.

**Table 1:** Summary statistics for all variables (60–89 years old)

Types	Variable names	Meanings and values	Mean	SD
Explained variable	MH	Mental health	3.125	0.668
Key explanatory variable	NMH	Neighborhood mental health	2.724	0.350
Instrumental variables	Neighborhood physical exercises	Neighborhood physical exercises (count, like NMH)	0.061	0.062
	Neighborhood amusement activities	Neighborhood amusement activities (count, like NMH)	0.153	0.096
Mediator variables	Surf the Internet	Surf the Internet within the past month = 1, otherwise = 0	0.071	0.258
	Volunteer activities	Yes = 1, No = 0	0.014	0.119
	Ln (gift money)	Ln (gift money)	3.478	3.674
Control variables	Gender	Male = 1, Female = 0	0.512	0.500
	Age	Age in 2018	68.154	6.177
	Marital status	With spouse = 1, otherwise = 0	0.789	0.408
	Educational attainment	uneducated = 1, Not graduated from primary school = 2, finish primary school = 3, Junior high school school = 4, Senior high school = 5, otherwise = 6	2.688	1.402
	Habitation	Agricultural = 1, otherwise = 0	0.721	0.449
	Ln(personal income)	Ln(total personal income)	7.307	3.009
	ADL	Activities of daily living	3.902	0.262
	IADL	Instrumental activity of daily living	3.493	0.338
	Family Doctor Services	Receive the paid family doctor services = 1, otherwise = 0	0.046	0.209
	Medical service satisfaction	Very bad = 1, bad = 2, neutral = 3, good = 4, vary good = 5	3.325	1.123
	Total of children	Number of all adult children	3.172	1.594
	Ln(Economic support)	Ln(Economic support from all children)	4.091	3.470
	Accompanying time from adult children	Accompanying time from all adult children	2.171	3.097

### Statistical analyses

#### OLS model

In this report, the neighborhood mental health (NMH) of older adults is considered the main explanatory variable, and the simple CES-D score is used as the proxy variable for the mental health of older adults, which is introduced into the model as an explained variable. However, because mental health is a continuous variable, OLS linear regression is adopted as the basic measurement model in this paper, as shown in Eq. 2:

2 $${MH}_{i}={\mathrm{\alpha }}_{0}+{\mathrm{\alpha }}_{1}{NMH}_{i}+\upbeta {\mathrm{X}}_{i}+{\upmu }_{i}$$

Where *i* is an individual; ${NMH}_{i}$is the core explanatory variable concerned in this paper, which is the NMH of individual* I*; MH_*i*_ is the mental health of individual *i*;${X}_{i}$ it is a control variable, including gender, age, marriage, education, residence, logarithm of annual income, ADL, IADL, number of children, economic support of children, accompanying time of children, satisfaction with medical services, and paid family doctor services; Coefficient α1 can reflect the degree of neighborhood health effect on the health of older adults, which is the key of this paper; α_0_ is the intercept term; β is the coefficient of the control variable; *u*_*i*_ is the random disturbance term.

#### IV-2SLS model

In the course of the research in this paper, two possible conditions lead to endogenous problems. First, important explanatory variables may be omission. Second, the life of older adults can’t be separated from their own groups, and their usual chat and behavior cannot help, showing their mental status, which may affect the mental status of their neighbors. To solve the problems caused by endogeneity, we introduce instrumental variables. This paper uses physical exercise and amusement as the instrumental variables of neighborhood mental health effects to solve the endogeneity problem caused by omitting important explanatory variables. As an instrumental variable, it must meet both the correlation and exogenous conditions. Physical exercise and amusement have an impact on the health of older adults who participated but have no impact on older adults who did not participate by meeting the two conditions of instrumental variables. We picked up two tool variables, neighborhood physical exercises and Neighborhood amusement activities, and used 2SLS to recheck the relationship between NMH and the mental health of older adults. The first step of 2SLS is to use NMH to regress the tool variables to gain the fitting value NMH* of NMH. The equation is as follows:

3 $${NMH}_{i}={\alpha }_{211}+{\alpha }_{212}{NFEs}_{i}+{\alpha }_{213}{NLAs}_{i}+{\beta }_{211}{Control}_{i}+{\varepsilon }_{21i}$$

4 $${MH}_{i}={\beta }_{221}+{\beta }_{222}{NMH}_{i}^{*}+{\beta }_{223}{Control}_{i}+{\varepsilon }_{22i}$$

We used the fitting value of NMH to complete the second-stage regression estimation of 2SLS (Eq. 4). Here, $${MH}_{i}$$ refers to the mental health,$$\alpha_{211}\;\mathrm{and}\;\beta_{221}\;\mathrm{are}\;\mathrm{the}\;\mathrm{constants},\;\alpha_{212}$$, $${\alpha }_{213}, {\beta }_{211}$$$$, {\beta }_{222}, {\beta }_{223}$$ are the coefficients, $${\varepsilon }_{21i}$$ and $${\varepsilon }_{22i}$$ are random disturbances.

#### KHB model

In order to further explore the mechanism of neighborhood mental effects on the mental health of older adults, this report adopts the KHB mediation effect analysis method and uses social contact, social participation, and social interaction as intermediary variables. Social contact is used to measure the scope of contact between older adults and their neighbors, and surfing the Internet is used as the proxy indicator. Volunteer activities are used as indicators to reflect social participation. Social interaction measures the closeness of the relationship between older adults and their neighbors. In China, we attach great importance to reciprocity, so this paper uses “gift money” as an indicator to reflect social interaction. We used the following equation:

5 $${MH}_{i}={\alpha }_{311}+{\alpha }_{312}{NME}_{i}+{\beta }_{311}{Control}_{i}+\gamma {{Mediator}_{i}+\varepsilon }_{31i}$$

6 $${MH}_{i}={\alpha }_{321}+{\alpha }_{322}{NME}_{i}+{\beta }_{321}{Control}_{i}{+\varepsilon }_{32i}$$

7 $$Residual={Mediator}_{i}-({\alpha }_{331}+{\alpha }_{331}{NME}_{i}+{\beta }_{331}{Control}_{i})$$

In Eq. 5, ${MH}_{i}$ refers to mental health, ${\alpha }_{311}$ is the constant, ${\alpha }_{312},{\beta }_{311},\gamma$ are coefficients, and ${\varepsilon }_{31i}$ refers to the residual. In Eq. 6,${\alpha }_{321}$ is a constant, ${\alpha }_{322}$ and ${\beta }_{321}$ are coefficients, ${\varepsilon }_{32i}$ represents the residual. Equation 7 refers to the extraction of information not contained in NME from the ${Mediator}_{i}$. Replacing the mediation variable of Eq. 8 with residual yields in the following formula:

8 $${MH}_{i}=\widetilde{{\alpha }_{311}}+\widetilde{{\alpha }_{312}}{NME}_{i}+\widetilde{\gamma }Residual+\widetilde{{\beta }_{311}}{Control}_{i}+{\varepsilon }_{41i}$$

The above formula further examines whether the difference between ${\alpha }_{322}$ and $\widetilde{{\alpha }_{312}}$ is significant; if the difference is significant, the mediating effect is established. If the mediating effect holds, we separately added the three mediating variables, social contact (surfing the Internet), social participation (Volunteer activities), and social interaction ("gift money"), and then the variables are added together to build the complete model.

## Results

### OLS regression analysis

Table 2 depicts the OLS model results. When controlling for variables such as the basic individual condition of the older adults, economic condition, family care, and public health policy, the NMH effect has a significant positive impact on the mental health of older adults, as shown in model I. The results of model II are obtained by adding the basic characteristics of older adults based on the NMH effect. Model III is the regression result of the NMH effect, the basic situation of older adults, and the economic situation of older adults. The results of model IV are based on the addition of family care to model III. Model V adds public medical policies based on model IV. Every unit of improvement in the NMH level will promote the mental health level of older adults by 0.233 units. Table 2 manifests that gender (Coef. = 0.170, 95% CI 0.141,0.200), marital status (Coef. = 0.108, 95% CI 0.072,0.144), education (Coef. = 0.047, 95% CI 0.036,0.058), ln(personal income) (Coef. = 0.010, 95% CI 0.005,0.015), ADL (Coef. = 0.487, 95% CI 0.406,0.568), IADL (Coef. = 0.284, 95% CI 0.229,0.339), total of children (Coef. = 0.017, 95% CI 0.007,0.028), ln(Economic support) (Coef. = 0.008, 95% CI 0.004,0.012), accompanying time of children (Coef. = 0.011, 95% CI 0.006,0.015), medical service satisfaction (Coef. = 0.070, 95% CI 0.058,0.083) have a significant positive impact on the health level of older adults. In contrast, age (Coef. = -0.005, 95% CI -0.008, -0.003), and habitation (Coef. = -0.064, 95% CI -0.098, -0.029) have a significant negative influence on the health level of older adults. However, family doctor services have no influence on the health of older adults. The OLS regression results of family doctor variables are insignificant because the number of older people participating in the project is small. Only 4.43% (440) of older adults in the sample used in this paper participated in the project, so the corresponding policy effect is not reflected.

**Table 2:** NMH on older adults (OLS regression model)

	( I)	(II)	(III)	(IV)	(V)
Neighborhood mental health	0.356^***^	0.246^***^	0.248^***^	0.238^***^	0.233^***^
	[0.315,0.397]	[0.201,0.291]	[0.205,0.291]	[0.194,0.282]	[0.190,0.277]
Gender		0.174^***^	0.163^***^	0.162^***^	0.170^***^
		[0.143,0.205]	[0.133,0.192]	[0.133,0.192]	[0.141,0.200]
Age		0.000	-0.003^***^	-0.006^***^	-0.005^***^
		[-0.002,0.002]	[-0.006,-0.001]	[-0.008,-0.003]	[-0.008,-0.003]
Marital status		0.106^***^	0.101^***^	0.105^***^	0.108^***^
		[0.068,0.145]	[0.065,0.137]	[0.069,0.141]	[0.072,0.144]
Educational attainment		0.048^***^	0.044^***^	0.042^***^	0.047^***^
		[0.036,0.060]	[0.033,0.055]	[0.031,0.053]	[0.036,0.058]
Habitation		-0.060^***^	-0.057^***^	-0.048^***^	-0.064^***^
		[-0.097,-0.024]	[-0.092,-0.022]	[-0.083,-0.013]	[-0.098,-0.029]
Ln(personal income)		0.017^***^	0.011^***^	0.011^***^	0.010^***^
		[0.011,0.022]	[0.006,0.016]	[0.006,0.016]	[0.005,0.015]
ADL			0.514^***^	0.507^***^	0.487^***^
			[0.432,0.597]	[0.425,0.589]	[0.406,0.568]
IADL			0.290^***^	0.290^***^	0.284^***^
			[0.235,0.345]	[0.235,0.345]	[0.229,0.339]
Total of children				0.016^***^	0.017^***^
				[0.005,0.026]	[0.007,0.028]
Ln(Economic support)				0.008^***^	0.008^***^
				[0.004,0.012]	[0.004,0.012]
Accompanying time from adult children				0.010^***^	0.011^***^
				[0.006,0.015]	[0.006,0.015]
Family Doctor Services					-0.048
					[-0.116,0.019]
Medical service satisfaction					0.070^***^
					[0.058,0.083]
Constant	2.155^***^	2.080^***^	-1.118^***^	-1.242^***^	-1.326^***^
	[2.040,2.270]	[1.864,2.295]	[-1.478,-0.758]	[-1.601,-0.883]	[-1.680,-0.973]
N	7713	7713	7713	7713	7713
Adj-*R*^2^	0.035	0.093	0.187	0.191	0.204

*Note*: *, **, and *** indicate significance at the 10%, 5%, and 1% statistical levels; 95% confidence interval in square brackets

### Endogenous problems

The instrumental variables selected in this article are the average physical exercise and amusement time of other older people in the community. The two-stage least squares estimation results obtained using the instrumental variable method are shown in Table 3. The first stage regression results reveal that physical exercise in other older adults (Coef. = 1.185, 95% CI 1.052,1.319) and amusement activity in other older adults (Coef. = 0.411, 95% CI 0.334,0.488) have a significant impact on NMH. It shows that the two tool variables used in this paper meet the correlation assumption. At the same time, we use a variety of statistical checkouts to judge the tool variables. The *P*-value (0.608) of Sargan statistics is greater than 0.1. The original assumption that the tool variable is exogenous is accepted, and the tool variable is qualified; it has nothing to do with the perturbation term. The underidentification test value is 441.955; statistical tests reject the original assumption that the tool variables are unrecognized at the 1% significance level. The weak identification test value is 282.728, greater than the critical value of 19.93, indicating a strong correlation between instrumental variables and endogenous variables [47, 48]. Furthermore, the weak instrument robust inference value was 11.32, reaching a significant level of 1%, which further indicated a strong correlation between instrumental variables and endogenous variables. Therefore, the assumption that instrumental variables are weakly identified is rejected. Table 3 depicts the report of three tests considering the instrumental variables used in this article. The second stage estimation results indicate that the estimation coefficient of the neighborhood health effect is 0.251, and it passes the test at the 1% significance level.

**Table 3:** NMH on the mental health of older adults (IV-2SLS)

Regression results in the second stage: y = mental health		
Variable	Coefficient	95% Confidence Interval
Neighborhood mental health	0.251***	[0.096,0.405]
Gender	0.172***	[0.141,0.203]
Age	-0.005***	[-0.008,-0.003]
Marital status	0.108***	[0.072,0.143]
Educational attainment	0.047***	[0.035,0.059]
Habitation	-0.060**	[-0.108,-0.012]
Ln(personal income)	0.010***	[0.005,0.015]
ADL	0.487***	[0.406,0.568]
IADL	0.284***	[0.230,0.339]
Family Doctor Services	-0.049	[-0.117,0.019]
Medical service satisfaction	0.070***	[0.058,0.083]
Total of children	0.016**	[0.003,0.030]
Ln(Economic support)	0.008***	[0.004,0.012]
Accompanying time from adult children	0.011***	[0.006,0.015]
Constant	-1.372***	[-1.896,-0.847]
Regression results in the first stage: y = neighborhood mental health		
Neighborhood physical exercises	1.185***	[1.052,1.319]
Neighborhood amusement activities	0.411***	[0.334,0.488]
Control variables	YES	YES
Under identification test	Kleibergen-Paap rk LM statistic: 441.955***	
Weak identification test	Kleibergen-Paap rk Wald F statistic: 282.728***	
Weak-instrument-robust inference	Anderson-Rubin Wald: 11.32***	

*Note*: *, **, and *** indicate significance at the 10%, 5%, and 1% statistical levels; 95% confidence interval in square brackets. The sample size is 7713, and the Centered R2 is 0.205

### Robustness check

1. Replace the dependent variable. First, mental health with life satisfaction was replaced and used O-logit regression. The conclusion indicated that the NMH (OR = 0.426, 95% CI 0.283,0.569) still had a significant positive impact on life satisfaction (Table 4, column 1). Second, mental health was replaced with physical health and used O-logit regression. Column 2 of Table 4 manifests that the NMH (OR = 0.201, 95% CI 0.201,0.477) still significantly impacts physical health. Third, mental health was replaced with a three-year self-assessment health change and used O-logit regression. The study proved that the NMH (OR = 0.168, 95% CI 0.022,0.313) has a significant positive impact on the three-year self-assessment health change of older adults (Table 4, column 3).
2. Replace the independent variable. The previous phase of this paper looked at the neighborhood effect within the community. Here we used county-level NMH to replace community-level NMH. Consistency in policy and resource allocation, similarity in traditional culture and customs, lesser economic and environmental differences, and ease of communication due to transportation development are factors that make communities within the same county similar in terms of policy, resources, culture, economy, and communication. Thus, the county-level neighborhood health effect and the community-level neighborhood health effect are correlated, and this study also confirms that the higher-level neighborhood mental health effect also positively and significantly affects the mental health of older adults. Due to privacy issues, CHARLS 2018 did not show a prefecture code. However, the practical survey of the CHARLS 2018 focused on 150 counties in 126 cities to substitute the city variable for the county variable (Using Eq. 1), the county-level NMH was calculated and regressed. The regression results in the fourth column of Table 4 showing that the county-level NMH (Coef. = 0.722, 95% CI 0.641,0.803) positively affects the mental health of older adults.
3. Different sample tests. Whether or not older adults are physically disabled, they are divided into two groups: Physical disability (239 samples) and non-disability (6861 samples), and the effect of NMH on the mental health of older adults is tested again. The results certify that the mental health of 239 older adults with physical disabilities was affected by the NMH effect (Coef. = 0.405, 95% CI 0.154,0.656) (Table 4, column 6), whereas the mental health of 6861 older people without physical disabilities was still affected by the NMH effect (Coef. = 0.220, 95% CI 0.174,0.266) (Table 4, column 6).

**Table 4:** Robustness tests of NMH

	Life satisfaction	Physical health	Three-year self-assessment health change	Count-level mental health	Physical disability	Non-disability
	O-logit: Replaced core explanatory variables			OLS: Replaced interpreted variable	OLS: Different sample sizes	
Neighborhood mental health	0.426***	0.339***	0.168**		0.405***	0.220***
	[0.283,0.569]	[0.201,0.477]	[0.022,0.313]		[0.154,0.656]	[0.174,0.266]
County neighborhood mental health				0.726***		
				[0.645,0.808]		
Control variables	YES	YES	YES	YES	YES	YES
N	7713	7713	7708	7713	239	6861
Pseudo/Adj-R^2^	0.043	0.064	0.037	0.223	0.217	0.197

*Note*: *, **, and *** indicate significance at the 10%, 5%, and 1% statistical levels; 95% confidence interval in square brackets. The O-logit model reports the odds ratio

### Heterogeneity analysis

In order to test whether the NMH has heterogeneity on the mental health of older adults, this paper conducts OLS regression for differences in gender, age, household registration, and residence, as shown in Table 5. We also tested for differences in gender, and the effect of NMH has a significant positive impact on both men and women (Female (Coef. = 0.276, 95% CI 0.209,0.343), Male (Coef. = 0.26, 95% CI 0.150,0.262)). The WTO classified older people into three stages: young seniors aged 60 to 74, those aged 75 to 89, and those with longevity aged 90 and above. Since the longevous is only 0.16% (12 persons), we will combine this part of the data into the old. The NMH effect has a positive impact on the young old and the old old, and the regression results are the young old (Coef. = 0.225, 95% CI 0.116, 0.334); the old old (Coef. = 0.248, 95% CI 0.201,0.295).

**Table 5:** Heterogeneity analysis of NMH by gender and age

	Male	Female	The young old	The old old
Neighborhood mental health	0.206^***^	0.276^***^	0.225^***^	0.248^***^
	[0.150,0.262]	[0.209,0.343]	[0.116,0.334]	[0.201,0.295]
Control variables	YES	YES	YES	YES
N	3949	3764	1276	6437
Adj-*R*^2^	0.159	0.193	0.186	0.204

*Note*: *, **, and *** indicate significance at the 10%, 5%, and 1% statistical levels; 95% confidence interval in square brackets

In order to further examine whether spouses have a heterogeneous effect on NMH, we divided the sample into two groups, spouse and without a spouse, and conducted OLS regression. Table 6 depicts the results. It was found that the effect of NMH had a significant positive influence on whether there was a spouse. The results showed with spouse (Coef. = 0.255, 95% CI 0.207,0.302) and without spouse (Coef. = 0.18, 95% CI 0.080,0.288). Simultaneously, we noticed that although the hukou system has been abolished in China, the actual urban–rural difference has always existed, so we divided the sample into urban and rural older adults based on their places of residence and performed the OLS regression analysis. The results showed with rural village (Coef. = 0.245, 95% CI 0.193,0.298) and urban community (Coef. = 0.235, 95% CI 0.157,0.312).

**Table 6:** Heterogeneity analysis of NMH by marriage and location

	With a spouse	Without a spouse	Rural village	Urban community
Neighborhood mental health	0.255^***^	0.184^***^	0.245^***^	0.235^***^
	[0.207,0.302]	[0.080,0.288]	[0.193,0.298]	[0.157,0.312]
Control variables	YES	YES	YES	YES
N	6082	1631	5560	2153
Adj-*R*^2^	0.198	0.187	0.184	0.192

*Note*: *, **, and *** indicate significance at the 10%, 5%, and 1% statistical levels; 95% confidence interval in square brackets

### Analysis of KHB mediation effect

This paper further used the KHB method to examine the intermediary effect, decomposing the total effect of NMH influence on the mental health of older adults into direct and intermediary effects, and calculated the size and contribution of NMH effect on the outcome variables through each intermediary path. We selected social contact, social participation, and social interaction as intermediary variables, surfing the Internet, and volunteer activities as proxy indicators of social contact and social participation, respectively, including gift cash income as proxy indicators of social interaction. surfing the Internet has a positive impact on the mental health of older adults. By going online, older adults can broaden their social circle, acquire information and knowledge, participate in online social interactions, and enrich their hobbies and recreational activities, which help reduce loneliness, enrich their spiritual life, and improve their quality of life and mental health. Participation in volunteer activities can enhance social connections, improve mental support networks and mental health; realize a sense of self-worth, increase self-esteem and sense of accomplishment; increase life satisfaction and positive experiences; increase mental adaptability and ability to cope with life's challenges; and maintain physical and mental vitality. Therefore, participation in volunteer activities is a way to promote the mental health of older adults. Gift cash is a traditional way to interact with friends and relatives, which can strengthen the connection between friends and relatives, provide mental support for the older adults, enhance their sense of security and improve their mental health. At the same time, gift exchange can also pass down neighborhood mutual aid and traditional cultural values, helping older adults have a stronger spiritual pillar in modern society. In addition, exchanging gifts can increase the sense of belonging among the older adults, reduce loneliness and mental stress, and improve mental health.

Table 7 presents the regression results are shown. Surf the Internet and NMH have significant positive effects (Coef. = 0.108, 95% CI 0.101,0.115), volunteer activities have a significant positive impact on the NMH (Coef. = 0.043, 95% CI 0.039,0.047), and gift money income has a significant positive impact on NMH (Coef. = 0.568, 95% CI 0.309,0.827).

**Table 7:** Influence of NMH on mediation mechanism variables

	Surfing the Internet	Volunteer activities	Ln (gift money)
Neighborhood mental health	0.108^***^	0.043^***^	0.568^***^
	[0.101,0.115]	[0.039,0.047]	[0.309,0.827]
Control variables	YES	YES	YES
N	7713	7713	7713
Adj-*R*^2^	0.416	0.291	0.070

*Note*: *, **, and *** indicate significance at the 10%, 5%, and 1% statistical levels; 95% confidence interval in square brackets

Based on the OLS benchmark model, we first calculated the intermediary effect of social contact separately, and the mediating effects of social activities were calculated independently. Finally, the mediating effect of social interaction was calculated individually. Then, these three intermediary variables are added to establish the whole model. Table 8 depicts the proportion of each intermediary variable that can be explained separately and the proportion that can be explained in the entire model. While surfing the Internet was put into the intermediary model alone, the total effect (Coef. = 0.356, 95% CI 0.314,0.398), direct effect (Coef. = 0.296, 95% CI 0.248,0.345), and indirect effect (Coef. = 0.06, 95% CI 0.035,0.084) were significant (p < 0.01). The total effect (Coef. = 0.356, 95% CI 0.314,0.398), direct effect (Coef. = 0.318, 95% CI 0.273,0.364), and indirect effect (Coef. = 0.038, 95% CI 0.019,0.056) were all significant (p < 0.01) when volunteer activities were put into the intermediary model alone. When gift money was put into the intermediary model alone, the total effect (Coef. = 0.356, 95% CI 0.314,0.398), direct effect (Coef. = 0.343, 95% CI 0.301,0.385), and indirect effect (Coef. = 0.013, 95% CI 0.008,0.018) were significant (p < 0.01). Surfing the Internet, volunteer activities, and gift money separately explained 16.77%, 10.57%, and 3.65% of the NMH effect. Simultaneously, the intermediary variables of surfing the Internet, volunteer activities, and gift money, the total effect of NMH (Coef. = 0.356, 95% CI 0.314,0.398), direct effect (Coef. = 0.281, 95% CI 0.232,0.330), and indirect effect (Coef. = 0.075, 95% CI 0.049,0.101) are significant (p < 0.01); the total effect is 1.266 times, 21.03% of the total effect comes from surfing the Internet, volunteer activities and gift money.

**Table 8:** Effect decomposition and comparison of KHB methods

	Surfing the Internet	Volunteer activities	Ln (gift money)		Surfing the Internet, voluntary activities & Ln(gift money)
Total effect	0.356***	0.356***	0.356***	Total effect	0.356***
	[0.314,0.398]	[0.314,0.398]	[0.314,0.398]		[0.314,0.398]
Direct effect	0.296***	0.318***	0.343***	Direct effect	0.281***
	[0.248,0.345]	[0.273,0.364]	[0.301,0.385]		[0.232,0.330]
Indirect effect	0.06***	0.038***	0.013***	Indirect effect	0.075***
	[0.035,0.084]	[0.019,0.056]	[0.008,0.018]		[0.049,0.101]
Separate Explanations	16.77%	10.57%	3.65%	Confounding ratio	1.266
Full model explanation	12.27%	5.24%	3.51%	Confounding percentage	21.03%

*Note*: *, **, and *** indicate significance at the 10%, 5%, and 1% statistical levels; 95% confidence interval in square brackets

## Discussion

Chinese communities have functions of psychological belonging, health support, social services, economic development, social governance, cultural education, environmental management, and democratic participation. When older adults live in the same community for a long time, various aspects of their lives intersect and are interrelated, leading to a strong presence of neighborhood effects. Based on the functions of psychological belonging and health support within the same community, this study investigates the impact of neighborhood mental health on the mental health of older adults.

### Key findings

In our study we found that there were three-way differences in the neighborhood mental health effect across different groups of older adults by gender, age, and presence of a spouse, respectively. First, differences in gender were found. This is similar to the findings of scholar Chrisinger [49], The neighborhood effect was greater for women than men this may be because among older adults of the same age, women are healthier than men [50] and can participate in more collective activities [51]. Conversely, women do more housework, go shopping more frequently than men, and have more opportunities to communicate with others [49]. The second is the age difference. Scholar Simran's research shows that the neighborhood effect does not increase but rather decreases as older adults age [52]. This is similar to our conclusion that the neighborhood mental health effect is greater for the young old than for the old old. This may have much to do with how China cares for older adults. Furthermore, after marrying, urban children no longer live with their parents to have independent space. After rural children get married, they leave their hometowns to work in cities, causing the phenomenon of empty nesters to become increasingly serious. As the age of the empty nest older adults increases, their participation in social activities will reduce the impact on their mental health [53–55]. The third is the difference between a spouse and without a spouse. Stahl noted in his study that community neighborhood perception was more strongly related to depression in older adults living alone, depression in older adults living with family members is unrelated [56]. This is contrary to our conclusion that the neighborhood mental health effect has a greater impact on older adults with spouses than those without spouses. And we believe that this difference, which may be due to two reasons. On the one hand, older adults with spouses can receive community information from their spouses even if they do not go out, which increases information transmission channels. On the other hand, older adults with spouses are more willing to participate in social activities [51]. The fourth is the difference in older adult residences. The effect of NMH on rural village is greater than that of urban community. According to Mr. Fei Xiaotong, the development of rural China is based on kinship and geographical relations [57]. In rural China, Due to the influence of traditional culture and settlement in rural China, it is easy for neighbors to have dual relations of blood and geography due to marriage. In urban areas, the acceleration of China's urbanization process has led to a large number of people from rural areas pouring into the city. Simultaneously, the population boom in urban areas has also broken the familiar neighborhood relations. Thus, the neighborhood relationship is closer than that of cities, and rural residents prefer to be together in their leisure time to play cards and chat. Therefore, the neighborhood effect in rural areas of China is more powerful than that in urban areas.

### What this study adds

We used three methods to examine whether the NMH has a positive impact on the mental health of older people: replacement independent variables, replacement dependent variables, and different sample tests. First, we used the method of replacing independent variables and a higher county-level NMH to replace the NMH (community level). The use of county-level administrative divisions in China has a long history, and such administrative divisions have been used so far. Therefore, in the same county, there are also the same historical and cultural backgrounds, living customs, and public policies. The county-level NMH and the community-level NMH correlate. The study confirmed that the higher level NMH effect will also significantly impact the mental health of older people.

Second, we used life satisfaction, self-rated health, and three-year self-assessment health change to substitute dependent variables. Generally, life satisfaction is positively related to the mental health of older adults [11], so we believe that life satisfaction can be used as a proxy for the mental health of older adults. Self-rated health refers to an individual's overall assessment of their historical physical indicators and mental status. The advantage of this method is to make up for the lack of mental health to some extent [43, 58]. CHARLS 2018 collected the self-assessment health information of older adults, so we used self-assessment health to replace mental health and changed it to the O-logit model. The results showed that the NMH effect also positively impacts the self-assessment health of older people. Furthermore, the mental health measured by cross-sectional data ignores the lag effect of historical health and the current periodic instability of mental health. We used the three-year self-assessment of health changes to alleviate these two problems. According to CHARLS, the overall health change trend of older people in the past three years was recorded. Therefore, we constructed the variable of health change in the past three years and used the O-logit model. The study found that the effect of NMH had a significant positive impact on older adults' self-assessment health change in the past three years.

Finally, we divided the total samples into two subsamples with large size differences to examine the robustness of the model in different sample sizes. The older adults were divided into two groups: physically disabled and non-disabled people. The NMH effect positively impacts the mental health of older adults in 6437 large samples with non-disabled participants. The findings support the positive impact of NMH effects on the mental health of older adults in 239 small samples with physical disabilities. Thus, it is concluded that the effect of NMH is always stable, whether physically disabled or not, and in a large or small sample.

### Strengths and limitations

In order to further explore the action mechanism of NMH for the mental health of old people, the intermediary variables of social contact, social participation, and social interaction are introduced. The results showed that surfing the Internet, volunteer activities, and gift money had significant effects on the mental health of older adults, indicating that strong social contact, social participation, and social interaction were conducive to the mental health of older adults. This is the same conclusion that Kail, Stevens, and other scholars have concluded that high levels of social engagement and social inclusion are important in supporting the physical and mental health of older adults to achieve productive and successful aging [59, 60]. And, we also believe that the reason may be that strong social participation and social integration can provide spiritual sustenance and self-worth affirmation for the older adults, which is conducive to the older adults' positive emotions. Thus, the mental health of older adults can be kept at a better level.

Notably, the present research has two defects. First, CHARLS historical survey did not involve the mental health of older adults, and we had to use cross-sectional data. Although we attempted to replace older adults' mental health with life satisfaction, self-assessment of health, and three-year health change to clarify the model, we must admit that these methods cannot compensate for the shortcomings of panel data models. Second, there are limitations to the mediating variables used in this paper. On the one hand, CHARLS conducted a survey in 2017 that revealed mobile Internet usage is significantly lower than the current level. Short videos such as TikTok have not yet emerged, and the mediating effect of mobile Internet will be more significant today. On the other hand, CHARLS did not ask questions about the community environment and neighborhood relations, so we used gift money as a proxy variable for social interaction. Therefore, the intermediary mechanism between mobile Internet and neighborhood mental health can be further explored in future research.

## Conclusion

China is at a critical stage of coping with the aging population and the healthy China strategy. Understanding the action mechanism of the NMH effects on the mental health of older adults has important implications for better promoting the construction of an aging community and an elderly-friendly society. Based on the 2018 CHARLS data, this report identified the internal relationship between the mental health of neighbors and the mental health of the older. The neighborhood mental effect increased by 1 unit, whereas the mental health of an older person increased by 0.251 units on average. Social contact, participation, and interaction are important channels for neighborhood mental health to improve the mental health of older adults. Further research on the heterogeneity of the neighborhood mental effect showed that the neighborhood mental effect has a greater influence on women than men, on the young old than the old, on older adults with a spouse than older adults without a spouse, and on rural villages than the urban community. Based on the empirical finding of this study, we recommended the following suggestions: First, we should promote the social participation of older adults through various forms of collective activities organized by communities. Various community activities increase the opportunities for older adults to join social activities. Older adults can enrich their lives, enjoy their emotions and promote their mental health by participating in activities. Second, the community or children encourage older adults to improve the use of intelligent networks. The correct use of intelligent networks can increase the channels for older adults to obtain information and expand the social scope of older adults so that older adults can break regional social barriers. Third, build a harmonious neighborhood culture. Mutual aid and friendly neighborhood relationship can promote interaction and communication between neighbors, enhance older adults' sense of trust and security in the living environment, and is beneficial to older adults' mental health.

## Data Availability

The data that support the findings of this study are available from Peking University, but restrictions apply to the availability of these data, which were used under license for the current study, and so are not publicly available. Data are however available from the authors upon reasonable request and with permission of Peking University.

## References

[1] NHC. National Health Commission of the People’s Republic of China (2022). China’s work on aging in the past decade has achieved remarkable results.

[2] NHC (2019). ranscript of the Special Press Conference of the National Health Commission of the People’s Republic of China on November 1, 2019.

[3] NHC (2022). Announcement of the Release of the “14th Five-Year Plan” for Healthy Ageing.

[4] World Health Organization. Office of World Health Reporting. The World health report : 1997 conquering suffering, enriching humanity: executive summary. Geneva: World Health Organization; 1997.

[5] Wilson WJ (2012). The Truly Disadvantaged: The Inner City, the Underclass, and Public Policy.

[6] Van Ham M, Boschman S, Vogel M (2018). Incorporating neighborhood choice in a model of neighborhood effects on income. Demography.

[7] Rengui G, Long Z, Zan H, Yangming H (2022). Neighborhood health effects on the physical health of the elderly: evidence from the CHRLS 2018. SSM - Population Health.

[8] Bahrainy H, Khosravi H (2013). The impact of urban design features and qualities on walkability and health in under-construction environments: the case of Hashtgerd New Town in Iran. Cities.

[9] Hinckson E, Cerin E, Mavoa S, Smith M, Badland H, Stewart T (2017). Associations of the perceived and objective neighborhood environment with physical activity and sedentary time in New Zealand adolescents. Int J Behav Nutr Phys Act.

[10] Liu Y, Wang R, Grekousis G, Liu Y, Yuan Y, Li Z (2019). Neighbourhood greenness and mental wellbeing in Guangzhou, China: What are the pathways?. Landsc Urban Plan.

[11] Liu J, Yang L, Xiao L, Tao Z (2022). perceived neighborhood environment impacts on health behavior, multi-dimensional health, and life satisfaction. Front Public Health.

[12] Murray ET, Nicholas O, Norman P, Jivraj S (2021). Life course neighborhood deprivation effects on body mass index: quantifying the importance of selective migration. IJERPH.

[13] Macintyre S, Ellaway A, Cummins S (2002). Place effects on health: how can we conceptualise, operationalise and measure them?. Soc Sci Med.

[14] Saelens BE, Handy SL (2008). Built environment correlates of walking. Med Sci Sports Exerc.

[15] Bakolis I, Murray ET, Hardy R, Hatch SL, Richards M (2023). Area disadvantage and mental health over the life course: a 69-year prospective birth cohort study. Soc Psychiatry Psychiatr Epidemiol.

[16] Li S-J, Luo Y-F, Liu Z-C, Xiong L, Zhu B-W (2021). Exploring strategies for improving green open spaces in old downtown residential communities from the perspective of public health to enhance the health and well-being of the aged. J Healthc Eng.

[17] de Sa E, Ardern CI (2014). Neighbourhood walkability, leisure-time and transport-related physical activity in a mixed urban–rural area. PeerJ.

[18] Frehlich L, Christie CD, Ronksley PE, Turin TC, Doyle-Baker P, McCormack GR (2022). The neighbourhood built environment and health-related fitness: a narrative systematic review. Int J Behav Nutr Phys Act.

[19] Chen X, Lee C, Huang H (2022). Neighborhood built environment associated with cognition and dementia risk among older adults: A systematic literature review. Soc Sci Med.

[20] Cunningham-Myrie CA, Theall KP, Younger NO, Mabile EA, Tulloch-Reid MK, Francis DK (2015). Associations between neighborhood effects and physical activity, obesity, and diabetes: The Jamaica Health and Lifestyle Survey 2008. J Clin Epidemiol.

[21] Mukherjee S, Frimpong Boamah E, Ganguly P, Botchwey N (2021). A multilevel scenario based predictive analytics framework to model the community mental health and built environment nexus. Sci Rep.

[22] Zheng Z, Liu W, Lu Y, Sun N, Chu Y, Chen H (2022). The influence mechanism of community-built environment on the health of older adults: from the perspective of low-income groups. BMC Geriatr.

[23] Jivraj S, Murray ET, Norman P, Nicholas O (2020). The impact of life course exposures to neighbourhood deprivation on health and well-being: a review of the long-term neighbourhood effects literature. Eur J Pub Health.

[24] Ruijsbroek A, Mohnen SM, Droomers M, Kruize H, Gidlow C, Gražulevičiene R (2017). Neighbourhood green space, social environment and mental health: an examination in four European cities. Int J Public Health.

[25] Abdul Manaf MR, Mustafa M, Abdul Rahman MR, Yusof KH, Abd Aziz NA (2016). Factors influencing the prevalence of mental health problems among Malay elderly residing in a rural community: a cross-sectional study. PLoS One.

[26] Heine C, Gong CH, Browning C (2019). Dual Sensory Loss, Mental Health, and Wellbeing of Older Adults Living in China. Front Public Health.

[27] Liu CP, Wang LJ (2017). A study of the impact of socio-economic status on the health of the elderly. Chin J Popul Sci.

[28] Dunkel Schetter C, Schafer P, Lanzi RG, Clark-Kauffman E, Raju TNK, Hillemeier MM (2013). Shedding light on the mechanisms underlying health disparities through community participatory methods. Perspect Psychol Sci.

[29] Ling C, Zhang A, Zhen X (2018). Peer effects in consumption among Chinese rural households. Emerg Mark Financ Trade.

[30] Oakes JM (2004). The (mis)estimation of neighborhood effects: causal inference for a practicable social epidemiology. Soc Sci Med.

[31] Manski CF (1991). Identification of Endogenous Social Effects: the Reflection Problem. Wisconsin Madison - Social Systems.

[32] Card D, Krueger AB (1998). School Resources and Student Outcomes. Ann Am Acad Pol Soc Sci.

[33] Moffitt RA, Comments T (2001). Policy Interventions, Low-Level Equilibria And Social Interactions. Social Dynamics.

[34] Paul ST (2004). School subsidies for the poor: evaluating the Mexican Progresa poverty program. J Dev Econ.

[35] Bobonis GJ, Finan F (2009). Neighborhood peer effects in secondary school enrollment decisions. Rev Econ Stat.

[36] Cherubal AG, Suhavana B, Padmavati R, Raghavan V (2019). Physical activity and mental health in India: a narrative review. Int J Soc Psychiatry.

[37] Wang H, Yang Y, You Q, Wang Y, Wang R (2022). Impacts of physical exercise and media use on the physical and mental health of people with obesity: based on the CGSS 2017 survey. Healthcare.

[38] Santini ZI, Meilstrup C, Hinrichsen C, Nielsen L, Koyanagi A, Koushede V (2020). Associations between multiple leisure activities, mental health and substance use among adolescents in denmark: a nationwide cross-sectional study. Front Behav Neurosci.

[39] Yoshida Y, Iwasa H, Ishioka Y, Suzukamo Y (2021). Leisure activity moderates the relationship between living alone and mental health among <scp>J</scp> apanese older adults. Geriatr Gerontol Int.

[40] Zhang C, Qing N, Zhang S (2021). The Impact of leisure activities on the mental health of older adults: the mediating effect of social support and perceived stress. J Healthc Eng.

[41] Dekker LH, Rijnks RH, Mierau JO (2021). The health potential of neighborhoods: a population-wide study in the Netherlands. SSM Population Health.

[42] Wang J, Liang C, Li K (2020). Impact of internet use on elderly health: empirical study based on Chinese general social survey (CGSS) Data. Healthcare.

[43] Zhang W, Wu YY (2017). Individual educational attainment, neighborhood-socioeconomic contexts, and self-rated health of middle-aged and elderly Chinese: exploring the mediating role of social engagement. Health Place.

[44] Hinata A, Kabasawa K, Watanabe Y, Kitamura K, Ito Y, Takachi R (2021). Education, household income, and depressive symptoms in middle-aged and older Japanese adults. BMC Public Health.

[45] Lu J, Zhang L, Zhang K (2021). Care preferences among Chinese older adults with daily care needs: individual and community factors. Res Aging.

[46] Ha SC (2022). Nam E.

[47] Kleibergen F, Paap R (2006). Generalized reduced rank tests using the singular value decomposition. J Econ.

[48] Stock JH, Yogo M (2002). Testing for Weak Instruments in Linear IV Regression.

[49] Chrisinger BW, Springfield S, Whitsel EA, Shadyab AH, Krok-Schoen JL, Garcia L (2022). The association of neighborhood changes with health-related quality of life in the women’s health initiative. IJERPH.

[50] Pachana NA, McLaughlin D, Leung J, McKenzie SJ, Dobson A (2011). The effect of having a partner on activities of daily living in men and women aged 82–87 years. Maturitas.

[51] Wang R, Chen H, Liu Y, Lu Y, Yao Y (2019). Neighborhood social reciprocity and mental health among older adults in China: the mediating effects of physical activity, social interaction, and volunteering. BMC Public Health.

[52] Godhwani S, Jivraj S, Marshall A, Bécares L (2019). Comparing subjective and objective neighbourhood deprivation and their association with health over time among older adults in England. Health Place.

[53] Wang R, Feng Z, Liu Y, Lu Y (2020). Relationship between neighbourhood social participation and depression among older adults: a longitudinal study in China. Health Soc Care Community.

[54] Zhang C, Zhu R, Lu J, Xue Y, Hou L, Li M (2018). Health promoting lifestyles and influencing factors among empty nesters and non-empty nesters in Taiyuan, China: a cross-sectional study. Health Qual Life Outcomes.

[55] Zhang C, Hou L, Zheng X, Zhu R, Zhao H, Lu J (2019). Risk factors of mental disorders among empty and non-empty nesters in Shanxi, China: a cross-sectional study. Health Qual Life Outcomes.

[56] Stahl ST, Beach SR, Musa D, Schulz R (2017). Living alone and depression: the modifying role of the perceived neighborhood environment. Aging Ment Health.

[57] Fei X (1992). From the Soil: The Foundations of Chinese Society.

[58] Yang F, Zhang J, Wang J (2018). Correlates of loneliness in older adults in Shanghai, China: does age matter?. BMC Geriatr.

[59] Kail BL, Carr DC (2020). Structural social support and changes in depression during the retirement transition: “i get by with a little help from my friends”. J Gerontol B Psychol Sci Soc Sci.

[60] Stevens M, Cruwys T, Haslam C, Wang V (2021). Social group memberships, physical activity, and physical health following retirement: a six-year follow-up from the English Longitudinal Study of Ageing. Br J Health Psychol.

